# Group A Beta-Hemolytic Streptococcus-Induced Tic-Like Movement Disorder in an Adult: A Case Report

**DOI:** 10.7759/cureus.28451

**Published:** 2022-08-26

**Authors:** Usman Ilyas, Zaryab Umar, Dana Lin

**Affiliations:** 1 Internal Medicine, Icahn School of Medicine at Mount Sinai, Queens Hospital Center, New York, USA; 2 Psychiatry, Creedmoor Psychiatric Center, New York, USA

**Keywords:** streptococcus tic, streptococcus adult movement, pandas, streptococcal chorea, streptococcal induced movement disorder

## Abstract

A tic is a sudden, repeated, quick, and uncontrollable movement of a part of the body. Tic disorders have a multifactorial etiology, including genetic predisposition, autoimmunity, and environmental factors. Movement disorders following streptococcal infection are rare and typically present in the pediatric population. We present a unique case of a 31-year-old female admitted with involuntary movements of the upper extremities of three weeks duration. Her movements start as twitching before progressing to one hand hitting the other or hitting her face. She had a strong urge before giving in to complete the action. However, the movements were partially distractable, with considerable overlap between clinical features of organic and functional tics. After a detailed workup, including a negative magnetic resonance imaging (MRI) of the brain with and without contrast, MRI of the spine, computed tomography of the chest, abdomen, and pelvis for neoplasia, as well as blood work for autoimmunity, infections, and paraneoplastic syndrome, the serology came back strongly positive for antistreptolysin O and antideoxyribonuclease B titers. Additionally, a detailed psychiatric assessment ruled out conversion disorder leading to a diagnosis of streptococcus-induced movement disorder. After a failed inpatient trial of aripiprazole, the plan included initiating deutetrabenazine with close outpatient neurology follow-up after discharge.

## Introduction

A tic is the sudden, repeated, quick, and uncontrollable movement of a part of the body [1]. Tics can involve any body part, such as the face, shoulders, hands, or legs [1]. Tics almost always begin in childhood, most often around ages four to six years, with the severity reaching its peak around 10 to 12 years of age [2]. The three tic disorders included in the fifth edition of the Diagnostic and Statistical Manual of Mental Disorders are Tourette’s syndrome, persistent (also called chronic) motor or vocal tic, and provisional tic disorder [3]. Tic disorders have a multifactorial etiology, including genetic predisposition, autoimmunity, and environmental factors [4]. Movement disorders following streptococcal infection are rare and typically present in the pediatric population [4]. Additionally, functional or psychogenic tic-like behaviors have also been described in the literature, providing significant difficulty in making the diagnosis. Here, we present a unique case of a 31-year-old female with involuntary movements of the extremities with strongly positive antistreptolysin O (ASO) and anti-deoxyribonuclease B (anti-DNase B) titers and no other identifiable cause for her symptoms.

## Case presentation

The patient is a 31-year-old female with no significant past medical history who presented with a chief complaint of progressively worsening involuntary movements of the upper extremities for three weeks. The patient endorses she was in her usual state of health when two months ago, symptoms started with an initial presentation of pressure behind the eyes without associated visual changes. She states that she saw a physician at the time who advised her to reduce her salt intake with subsequent improvement of symptoms. However, she then developed jaw pain and abnormal movements in the upper extremities. Upper extremity movements initially started as twitching and then progressed to using one hand to punch the palm of her other and then hitting her face. The patient has not noticed any patterns or triggers to these movements but does endorse an urge before giving in to complete the action. The patient has previously seen a neurologist and received a prescription for alprazolam and trazodone, which she does not take. Previously, the patient had completed an entire course of treatment for chlamydia, trichomonas, and bacterial vaginosis. The patient also endorses having been told she needed a lumbar puncture but was unsure of its reason. Of note, the patient works at a mental health facility and denies exposure to animals, sick contacts, and toxins. In addition, the patient denies a history of drug use and smoking and drinks socially. The patient also denies any new-onset stressors but does cry during the interview endorsing that upper extremity movements impair her ability to function during day-to-day activities, including caring for her children. Family history includes stroke and ovarian cancer on the maternal side and unknown family history for the father. The patient denied headaches, fever, chills, chest pain, shortness of breath, cough, palpitations, nausea, vomiting, or changes in bowel and urinary habits. 

On physical exam, the patient, with a body mass index of 38 kg/m2, sleeps in no acute distress or movement. Upon waking, the patient progressively develops the tic-like motions she previously described throughout the interview. The motion looks to begin as a tremor in her right arm that progressively develops into more complex movements such as hitting the opposite palm with her first and eventually hitting her face with one arm. The physical exam was otherwise unremarkable except for hypopigmentation on the left temple, which the patient endorses as a birthmark. The patient can be temporarily distracted or calmed, which subsides movements. Of note, the patient also does not have tic-like movements during sleep.

The initial differential diagnosis list included anti-N-methyl-D-aspartate-receptor (anti-NMDAR) encephalitis, Wilson's disease, Huntington's disease, tic disorder, an adult variant of pediatric autoimmune neuropsychiatric disorders associated with streptococcal infections (PANDAS), and psychogenic or functional tics. On admission, lab results were unremarkable (Table 1).

**Table 1:** Labs obtained at the time of admission

Lab	Patient's value	Reference range and units
Hemoglobin	12.6	12.0-16.0 g/dL
White blood cell count	7.11	4.80-10.80 x10(3)/mcL
Platelets	329	150-450 x10(3)/mcL
Sodium	142	136-145 mmol/L
Potassium	3.8	3.5-5.1 mmol/L
Magnesium	2.10	1.60-2.60 mg/dL
Bicarbonate	23	22-29 mmol/L
Blood urea nitrogen	13	6-23 mg/dL
Creatinine	0.82	0.50-1.20 mg/dL
Alanine transaminase (ALT)	17	0-33 U/L
Aspartate transaminase (AST)	22	5-32 U/L
Alkaline phosphatase	63	35-104 U/L
Total bilirubin	0.6	0.0-1.2 mg/dL
Thyroid-stimulating hormone (TSH)	0.31	0.27-4.20 uIU/mL
Free T4	1.2	0.9-1.8 ng/dL
human chorionic gonadotrophin (hCG) quantitative	<0.5	<= 5.0 mIU/mL
Vitamin B12	1176	232-1245 pg/mL

**Figure 1:**
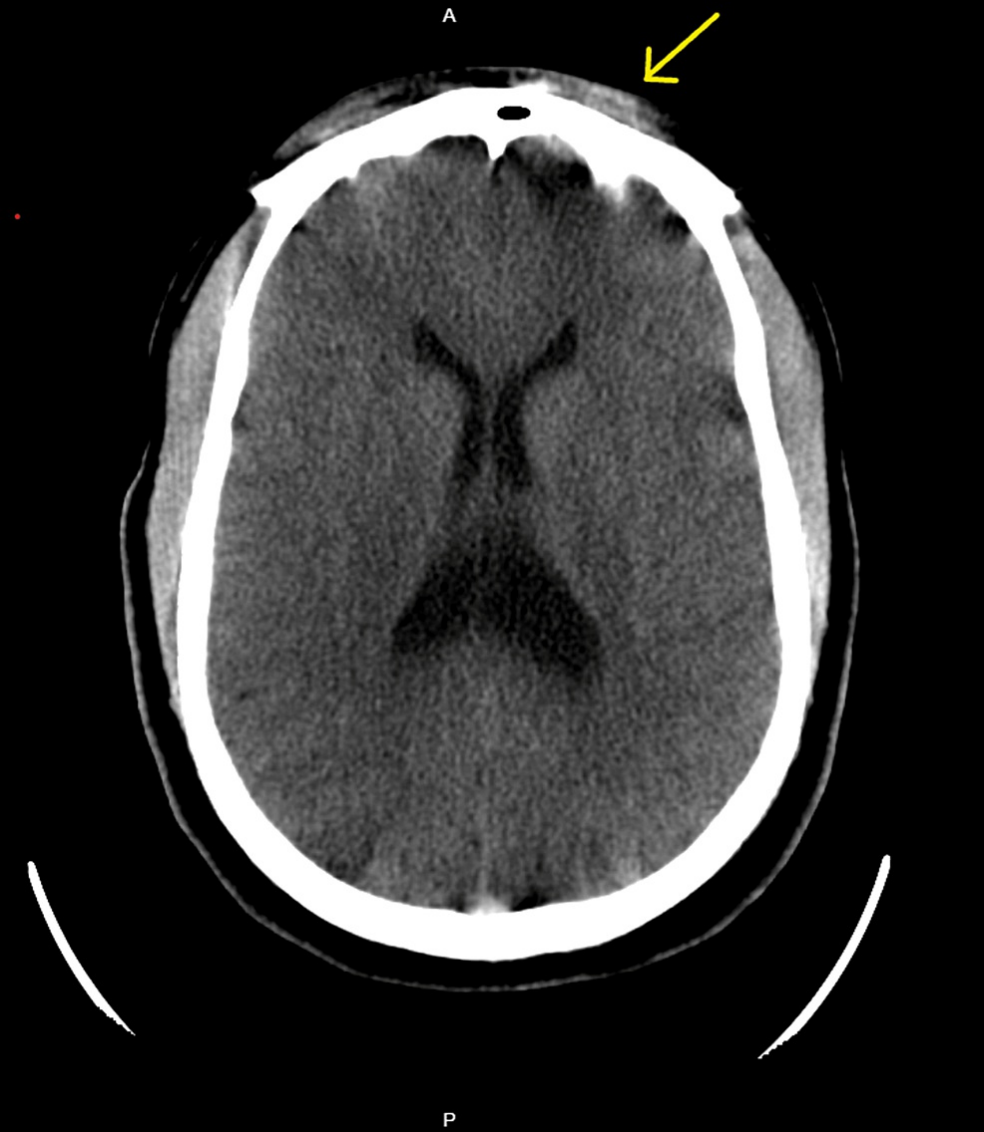
CT head shows a mild left frontal subcutaneous soft tissue swelling suggestive of a contusion or hematoma.

Imaging studies showed some motion artifacts as the patient had tic-like movements during imaging studies despite administration of lorazepam, which partially relieved symptoms. In addition, the head's computed tomography (CT) scan (Figure 1) shows mild left frontal subcutaneous soft tissue swelling suggestive of a contusion or hematoma, likely from trauma due to uncontrollable upper extremity movements. Otherwise, there were no significant findings. 

**Figure 2:**
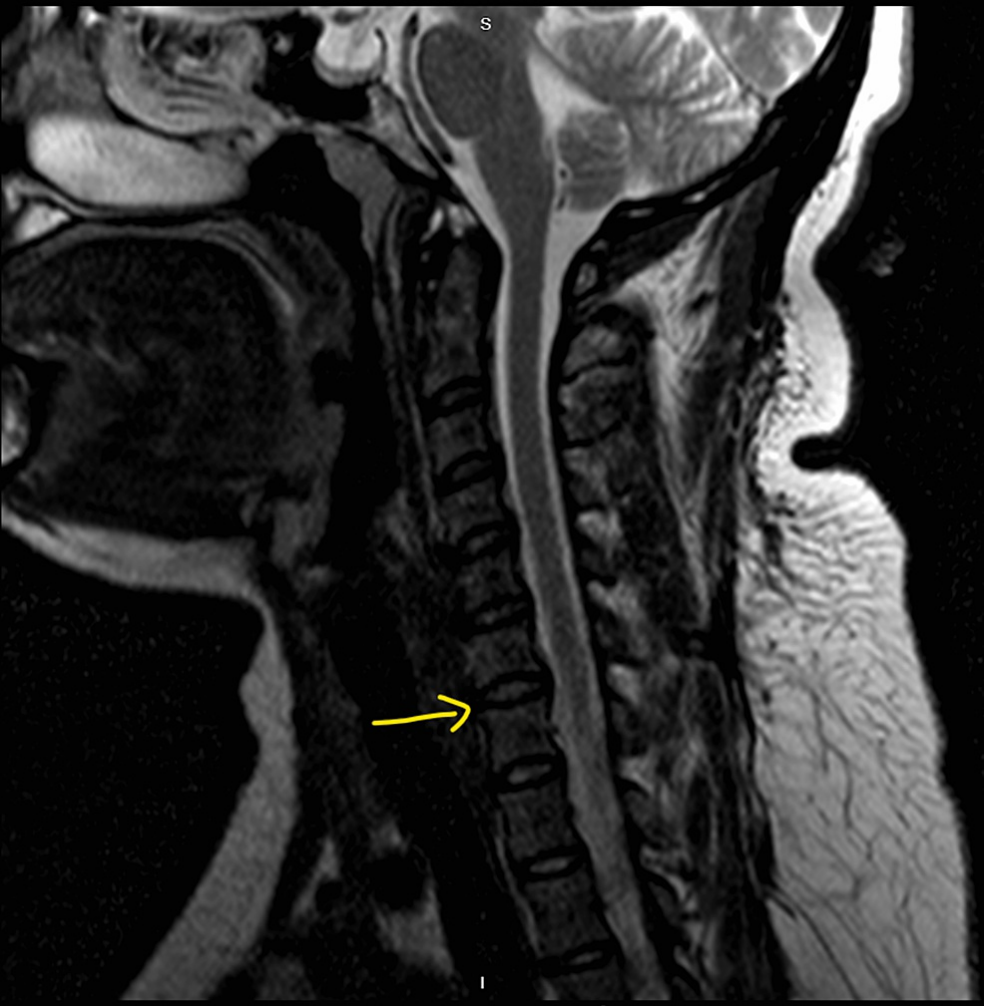
MRI of the cervical spine shows a small central disc protrusion between C4 and C5.

Magnetic resonance imaging (MRI) of the brain also showed no significant findings. However, MRI C-spine shows small central disc protrusion between C4 and C5, with trace ventral cord effacement (Figure 2). 

CT scan of the abdomen and pelvis showed a left ovary measuring 4.2 by 3.8 cm and a right ovary measuring 3.5 by 3.7cm. Both contained normal-appearing follicles with no evidence of intra-abdominal malignancy. The patient tested negative for human immunodeficiency virus, syphilis, antinuclear antibodies, anti-neuromyelitis optica antibody, anti-myelin oligodendrocyte glycoprotein antibody, anti-NMDA antibody, anti-Hu antibody, anti-Yo antibody, anti-Lyme antibody, elevated serum copper, and elevated 24-hour urine copper. Hepatic function panels were within normal limits. ASO and Anti-DNAse B titers were reactive to 553 and 181, respectively (Table 2). 

**Table 2:** Further workup is unremarkable apart from strongly positive anti-streptococcal antibodies.

Lab	Patient’s value	Reference range and units
Erythrocyte sedimentation rate	36	0-20 mm/hr
high sensitivity C-Reactive Protein	15.4	<=5.00 mg/L
Anti-Streptolysin O titer	553	0-199 IU/mL
Anti-deoxyribonuclease B titer	181	0-120 IU/mL
Human immunodeficiency virus antigen-antibody screen	Non Reactive	Non Reactive
Treponema pallidum antibody screen	Negative	Negative
Lyme total antibody immunoglobulin G/M	0.21	0.01-0.89
Ceruloplasmin	29	16-45 mg/dL
Copper, 24-hour urine	26	3-35 ug/24 hr
Ferritin	132	15-150 ng/mL
Antinuclear antibody screen	Negative	<1:80
anti-Yo (Purkinje Cell) antibody screen	Fluorescence Noted	
anti-Yo antibody western blot	Negative	Negative
anti-Hu antibody screen	Fluorescence Noted	
anti-Hu Antibody western blot	Negative	Negative
Neuromyelitis Optica antibody	<1.5	0.0-3.0 U/mL
Myelin oligodendrocyte glycoprotein reflex to titer	Negative	Negative
N-methyl-D-aspartate immunoglobulin G antibody	<1:10	<1:10
Drug screen qualitative	Benzodiazepine (Positive)	Negative
Tetrahydrocannabinol urine qualitative	Negative	Negative

The patient started on aripiprazole 5 mg per psychiatry, which showed no improvement in symptoms. The patient was discharged on deutetrabenazine, beginning at 12 mg daily, titrating up to 6 mg weekly to reach 42 mg split between two doses. 

## Discussion

Neuropsychiatric and behavioral disorders, including obsessive-compulsive disorder (OCD), Tourette Syndrome, and tic disorder, have a multifactorial etiology including genetic predisposition, autoimmunity, and environmental factors (e.g., pre and perinatal difficulties, infections, stress-inducing events) [4]. However, growing evidence suggests that they occur partly due to postinfectious autoimmune phenomena. The original hypothesis was proposed in the 1980s, when an outbreak of streptococcal tonsillitis in Rhode Island, United States of America, was associated with a 10-fold increase in the incidence of motor tics (without chorea) in the region [5]. Mell et al. concluded in a large case-control study that streptococcal infection seemed to double the risk of the first diagnosis of disorders as mentioned above within the first three months after infection and multiple infections seemed to approximately triple the chance for a first diagnosis within 12 months [6]. Other reported disorders following group A beta-hemolytic streptococcus (GABHS) infection include Sydenham chorea (SC), PANDAS, encephalitis lethargica, post-streptococcal acute disseminated encephalomyelitis, and adult-onset movement disorders [7]. In movement disorders, patients typically have dystonia, tremor, stereotypies, opsoclonus, catatonia, paroxysmal choreoathetosis, tics, parkinsonism, myoclonus, ataxia, and chorea [8].

Acute rheumatic fever and SC are two recognized entities that occur as post-streptococcal disease phenomena. Apart from involuntary movements, SC patients have associated neurologic and psychiatric symptoms, with some studies reporting up to 80% of the patients meet the diagnostic criteria for OCD [9]. Similarly, neuropsychiatric changes can be seen in patients with rheumatic fever but without chorea [9]. Therefore, considering there might be patients with neuropsychiatric symptoms related to GABHS without chorea, a relationship between autoimmunity and behavioral symptoms in patients with OCD and tics was proposed, coining a new clinical entity known as the PANDAS [9]. The five diagnostic criteria of PANDAS are 1) the presence of OCD and/or tics, 2) onset between three years of age and puberty, 3) abrupt onset of symptoms and/or course with recurrent exacerbations and remissions, 4) abnormal neurologic examination without frank chorea and 5) temporal association of symptom exacerbation with streptococcus infection [10]. PANDAS is mainly a pediatric disease with the age of onset ranging from three years to the beginning of puberty. However, this disorder has variants, including an adult-onset variant, a dystonic variant, a myoclonic variant, and a chronic PANDAS variant [9-10]. As noted above, recurrent exacerbation and remissions are required to make a diagnosis, which might be the case in our patient on subsequent follow-up. The onset of this disease is abrupt, with a slow and gradual resolution over weeks or months, with possible relapses due to new streptococcal infections [10]. Neuropsychiatric symptoms include tics, OCD, difficulty reading, attention deficit disorder, hyperactivity, depression, anxiety, emotional lability, sleep disorders, eating disorders, autism, and paroxysmal dyskinesias [10].

The pathophysiology involves a unique bacterial strain with a specialized M protein that induces an immune response by forming cross-reacting antibodies in a susceptible host with genetic factors contributing to autoimmunity [4]. Repeated infections, in turn, cause a robust T helper 17 cellular and humoral immune response [4]. In addition, the antibodies cross-react with antigens, currently unknown, enriched in basal ganglia. The presence of these antibodies in the serum supports the hypothesis that the condition's pathophysiology occurs via molecular mimicry [11]. In most cases, the precipitating infection typically occurs two to three weeks before the onset of the neurological disease. GABHS is children's most common bacterial cause of acute pharyngitis [12]. Clinically, it is difficult to differentiate between viral and bacterial infection, leading to overtreatment with antibiotics [12].

A rapid strep test successfully diagnoses and treats streptococcal pharyngitis in a pharyngeal swab [12]. However, although rapid strep testing and/or throat swab culture is the standard of care for acute pharyngitis, they are of limited utility in post-streptococcal disease since symptoms can follow weeks after throat infection when the throat culture would be negative and pharyngeal infection is not the only source of GABHS exposure [9]. This was also seen in our case as our patient presented two months after the onset of symptoms. Recent streptococcal infection can also be documented by increased anti-streptococcal antibodies including ASO (75% sensitive, 84% specific), anti-streptokinase (34% sensitive, 85% specific) and anti-DNAse B (70% sensitive, 99% specific) [12]. In our case, both ASO and anti-DNAse B were well above the cut-off value, which in combination, according to Blyth et al., is the most sensitive and specific method for confirming post-streptococcal disease [13]. The most common immunologic abnormality in patients with an autoimmune neuropsychiatric disorder associated with streptococcal infection is anti-basal ganglia antibodies detected in serum and/or cerebrospinal fluid [9]. They are seen with a much higher incidence in patients with PANDAS than those with uncomplicated streptococcal pharyngitis [9]. We could not perform cerebrospinal fluid analysis as the patient declined the procedure. The presence of antibodies directed against caudate and putamen was significantly higher in a cohort of children with new onset movement disorders (Tourette's syndrome, chronic motor or vocal tics, chorea, choreiform movements) and new onset OCD or pervasive obsessive-compulsive symptoms than in clinical controls without such symptoms [14].

Neuroimaging is usually normal in post-streptococcal central nervous system disorder or may show incidental findings [5]. Inflammatory changes in basal ganglia are occasionally seen [5]. Volumetric studies have shown an enlargement of the caudate and putamen during the acute phase of illness, with single positron emission computed tomography studies showing hyper or hypo metabolism in the basal ganglia [5]. These changes are usually reversible; however, persistent changes indicative of irreversible damage have been documented in the literature [5]. Additionally, another study compared 34 cases of PANDAS with 82 healthy controls and found that the average sizes of caudate, putamen and globus pallidus were significantly greater in the PANDAS group [15]. However, the size of the thalamus and total cerebrum was similar in both study groups [15]. In contrast, quantitative MRI in patients with idiopathic Tourette syndrome/OCD has reported decreased basal ganglia, frontal white matter, and corpus callosum volume [9]. Therefore, quantitative MRI can potentially differentiate between idiopathic/chronic Tourette syndrome/OCD and PANDAS [9].

Per current guidelines, penicillin is used during the acute phase after confirming streptococcal infection by a throat swab [15]. For people sensitive to penicillin, erythromycin is the best alternative [12]. Although there are no controlled studies regarding prophylactic treatment with penicillin to prevent further recurrence with exacerbations, preventive therapy has been postulated in literature [10]. In addition, selective serotonin reuptake inhibitors, neuroleptics, and cognitive behavioral therapy are used for tics and OCD [10]. Although immune therapies such as steroids, immunoglobulin, and plasma exchange have been successfully used in streptococcal-induced movement disorders until further studies document their benefit, the side effects of these therapies preclude their routine use [12].

There are several limitations to our study. First, since our patient presented two months after the onset of symptoms, we cannot definitively establish the temporal relationship between the movement disorder and GABHS. She had considerable overlap between the symptoms and signs of organic and functional/psychogenic tics, providing diagnostic difficulty. In addition, after discharge, she was lost to follow-up; therefore, subsequent progression of symptoms could not be studied, including the development of any neuropsychiatric symptoms and exacerbation or remission of movements.

## Conclusions

Our case report highlights the importance of a thorough history, including inquiring about past infections and investigations to look for autoimmune and infectious etiologies, including ASO and positive DNAse B titers, in adult patients with new onset movement disorders and no other identifiable etiology and risk factors. Further research is mandatory to investigate the incidence of movement disorders in the adult population after a streptococcal infection and the diagnostic approach and treatment modalities needed to manage such patients.
